# Quantifying effects of snow depth on caribou winter range selection and movement in Arctic Alaska

**DOI:** 10.1186/s40462-021-00276-4

**Published:** 2021-09-22

**Authors:** Stine Højlund Pedersen, Torsten W. Bentzen, Adele K. Reinking, Glen E. Liston, Kelly Elder, Elizabeth A. Lenart, Alexander K. Prichard, Jeffrey M. Welker

**Affiliations:** 1 Department of Biological Sciences, University of Alaska Anchorage, Anchorage, AK 99508 USA; 2 Cooperative Institute for Research in the Atmosphere, Colorado State University, Fort Collins, CO 80523 USA; 3 Alaska Department of Fish and Game, Fairbanks, AK 99701 USA; 4 US Forest Service, Rocky Mountain Research Station, Fort Collins, CO 80526 USA; 5 ABR, Inc.—Environmental Research & Services, Fairbanks, AK 99708 USA; 6 Ecology and Genetics Research Unit, University of Oulu, 90014 Oulu, Finland; 7 UArctic, University of the Arctic, 96101 Rovaniemi, Finland

**Keywords:** Arctic Alaska, Barren-ground caribou, Migration, Movement ecology, *Rangifer tarandus*, Integrated step selection analysis, Snow depth, SnowModel, Ungulate, Winter range

## Abstract

**Background:**

Caribou and reindeer across the Arctic spend more than two thirds of their lives moving in snow. Yet snow-specific mechanisms driving their winter ecology and potentially influencing herd health and movement patterns are not well known. Integrative research coupling snow and wildlife sciences using observations, models, and wildlife tracking technologies can help fill this knowledge void.

**Methods:**

Here, we quantified the effects of snow depth on caribou winter range selection and movement. We used location data of Central Arctic Herd (CAH) caribou in Arctic Alaska collected from 2014 to 2020 and spatially distributed and temporally evolving snow depth data produced by SnowModel. These landscape-scale (90 m), daily snow depth data reproduced the observed spatial snow-depth variability across typical areal extents occupied by a wintering caribou during a 24-h period.

**Results:**

We found that fall snow depths encountered by the herd north of the Brooks Range exerted a strong influence on selection of two distinct winter range locations. In winters with relatively shallow fall snow depth (2016/17, 2018/19, and 2019/20), the majority of the CAH wintered on the tundra north of the Brooks Range mountains. In contrast, during the winters with relatively deep fall snow depth (2014/15, 2015/16, and 2017/18), the majority of the CAH caribou wintered in the mountainous boreal forest south of the Brooks Range. Long-term (19 winters; 2001–2020) monitoring of CAH caribou winter distributions confirmed this relationship. Additionally, snow depth affected movement and selection differently within these two habitats: in the mountainous boreal forest, caribou avoided areas with deeper snow, but when on the tundra, snow depth did not trigger significant deep-snow avoidance. In both wintering habitats, CAH caribou selected areas with higher lichen abundance, and they moved significantly slower when encountering deeper snow.

**Conclusions:**

In general, our findings indicate that regional-scale selection of winter range is influenced by snow depth at or prior to fall migration. During winter, daily decision-making within the winter range is driven largely by snow depth. This integrative approach of coupling snow and wildlife observations with snow-evolution and caribou-movement modeling to quantify the multi-facetted effects of snow on wildlife ecology is applicable to caribou and reindeer herds throughout the Arctic.

**Supplementary Information:**

The online version contains supplementary material available at 10.1186/s40462-021-00276-4.

## Background

### Caribou as a snow-adapted species

Snow dominates Arctic landscapes for more than two thirds of the year [1], and therefore, snow, in its multiple forms, affects *Rangifer* species residing in northern regions globally. Caribou (*Rangifer tarandus *ssp.) are chionophiles; their morphology, physiology, and behavior evolved to enable survival in snowy winter environments [2–5]. Relatively long legs and low “foot loadings” are unique adaptations that help *Rangifer* cope with snow by providing efficient locomotion and establishment of feeding craters in snowy conditions [6]. Skin that is well-insulated by woolly fur overlaid with hollow guard hairs [7] provide extreme insulation and protection from wind [8, 9]. In addition to their sharp edges, caribou hooves also have microstructures of high skid and abrasion resistance, which allow caribou to travel on icy surfaces [10–13]. These traits enable caribou to remain mobile year-round, even during winters with extremely deep snow [14]. Consequently, their existence in the Arctic has been sustained for more than 50,000 years [5, 15, 16].

Despite the specialization of caribou to snowy conditions, observational research indicates that snow significantly affects caribou winter ecology [17]. For example, snow characteristics determine forage accessibility (e.g., [3, 4, 18]), energy expenditure in locomotion [6], daily activity budget (i.e., the amount of time spent cratering, resting, and moving [19]), and migration timing (e.g., [20]). The energetic costs and benefits related to these processes and activities likely influence where caribou spend the winter and their movement paths through snow-covered landscapes. However, the degree of control that snow exerts on caribou movement patterns and rates across different spatial scales throughout winter, and how that may vary among herds and among individuals, has received limited attention. Pruitt [3] recognized the large spatial and temporal variations in snow characteristics across caribou winter ranges. He argued that for the purpose of explaining caribou winter-range conditions, *“...conventional snow data as reported by the existing net of meteorological observatories are not only insufficient but may actually be misleading”*. Past studies have typically relied on snow field observations or snow remote sensing datasets that can be incompatible with the dynamic nature of wildlife movement data in terms of temporal frequency, spatial resolution and coverage, or the snow variables being represented; often, these datasets do not characterize the most ecologically meaningful snow properties at the most appropriate scales for wildlife applications [21]. While Pruitt advocated for the importance of incorporating spatiotemporal snow variability in caribou studies over 60 years ago, more recently Boelman et al. [21] argued it remains a persistent issue and data-gap in wildlife ecology. Here, we present snow datasets that can be integrated with spatiotemporally dynamic caribou movement data. The winter environmental modeling tools we used, SnowModel (presented in section 2.2.2; [22]), are capable of producing snow information that captures caribou- and other wildlife-relevant snow properties and their evolution across space and time; here, we utilized snow depth. With such information, it is now possible to more deeply investigate and quantify the role of snow in caribou winter ecology [21].

### Fall migration cues

Most barren-ground caribou conduct fall and spring migrations that bracket the Arctic winter; these can represent the longest-distance terrestrial migrations on the planet [23]. For migratory species, the timing and destination of these long, directed movements can be correlated to environmental cues such as vegetation phenology, photoperiod, previously-used migration trails, snow accumulation and melt, or temperature (e.g., [19, 20, 24–26]). An animal perceives cues from its surrounding environment that are indicative of conditions at different locations and/or in the future, which may be suitable for individuals to strategically carry out life-cycle events, such as migration, with minimal risk and maximum benefit [27, 28]. The perception of these cues affects an animal’s decision to initiate preparatory steps for migration, and hence, affects the seasonal timing of this life-cycle event [27].

Snow conditions can be a significant trigger for migratory species inhabiting seasonally variable environments (e.g., [29–31]). For instance, the first heavy snowfall after rut can trigger fall migration of caribou [32, 33]. In addition to initiating migration, fall environmental cues may influence selection of winter range location. Based on mostly anecdotal evidence, this appears to be the case among Arctic Alaska caribou herds, for which the first snowfall likely acts as a stimulus for fall migration [34, 35], and the cues for selection of winter range location likely include thresholds of snow depth, timing of first snowfall event, and olfactory sensing of lichen below the snow [36]. In the past, snow data at adequate spatial and temporal resolutions and extents, to capture changes in snow conditions that may trigger such behaviors, have been non-existent or inadequate [21] and, therefore, quantification of the cueing effect of snow has been limited. Such spatially and temporally explicit snow data, describing, e.g., snow accumulation, need to: 1) cover the entire migration route, including initiation location, migration path, stopover sites, and finally, winter range; 2) have adequate temporal frequency and detail to match the temporal scale at which a definitive change in caribou movement behavior is detected (e.g., daily); and 3) contain spatial and temporal information at sufficient detail to realistically capture the variability in snow accumulation within the initiation location of each individual caribou’s migration. Until now, such dynamic, spatially and temporally comprehensive snow information has been largely unavailable for wildlife applications [21].

### Winter range location and winter movement

At a regional scale, caribou wintering locations can vary considerably from year to year and include tundra and boreal forest habitats [19, 37–39]. For many herds across North America, their precise annual winter range location may depend largely on snow conditions and snow’s control on forage accessibility [3, 11, 40, 41]; winter forage distribution, particularly of lichen species [42–46]; fire history [42, 47]; and coincident predator abundance [33, 48].

At a landscape scale within their winter range, field observations suggest that snow depths tolerated by caribou differ between tundra and boreal forest habitats [4, 49]. However, in general, caribou select for forage areas with shallow and/or soft snow cover at both the landscape and patch scales (i.e., within tens of meters; [3, 4, 18, 36, 50, 51]). In tundra habitats, this selection typically results in winter movement patterns where caribou avoid depressions in the landscape, e.g., cut banks of rivers that are more likely full of deep, wind-compacted snow, and instead stay on snow-free ground, such as rounded hilltops with wind-swept slopes where ablation (sublimation and wind erosion) has significantly reduced the snowpack [11]. Caribou wintering in a boreal forest habitat typically move single-file through the characteristically deep, but soft, snow cover [52]; crater for forage below the snowpack or feed on arboreal lichen, which is more easily accessed than terrestrial lichen when the snowpack is deep [53]; and they may rest on lakes or seek out tree-less ridges that enable better detection of incoming predators [3, 48]. Such selection patterns translate into an ideal caribou winter range being characterized by snow conditions that provide easy access to forage, minimize energy costs related to mobility [54], and possibly enhance predator avoidance [55, 56].

### The Central Arctic Caribou Herd

**Fig. 1:**
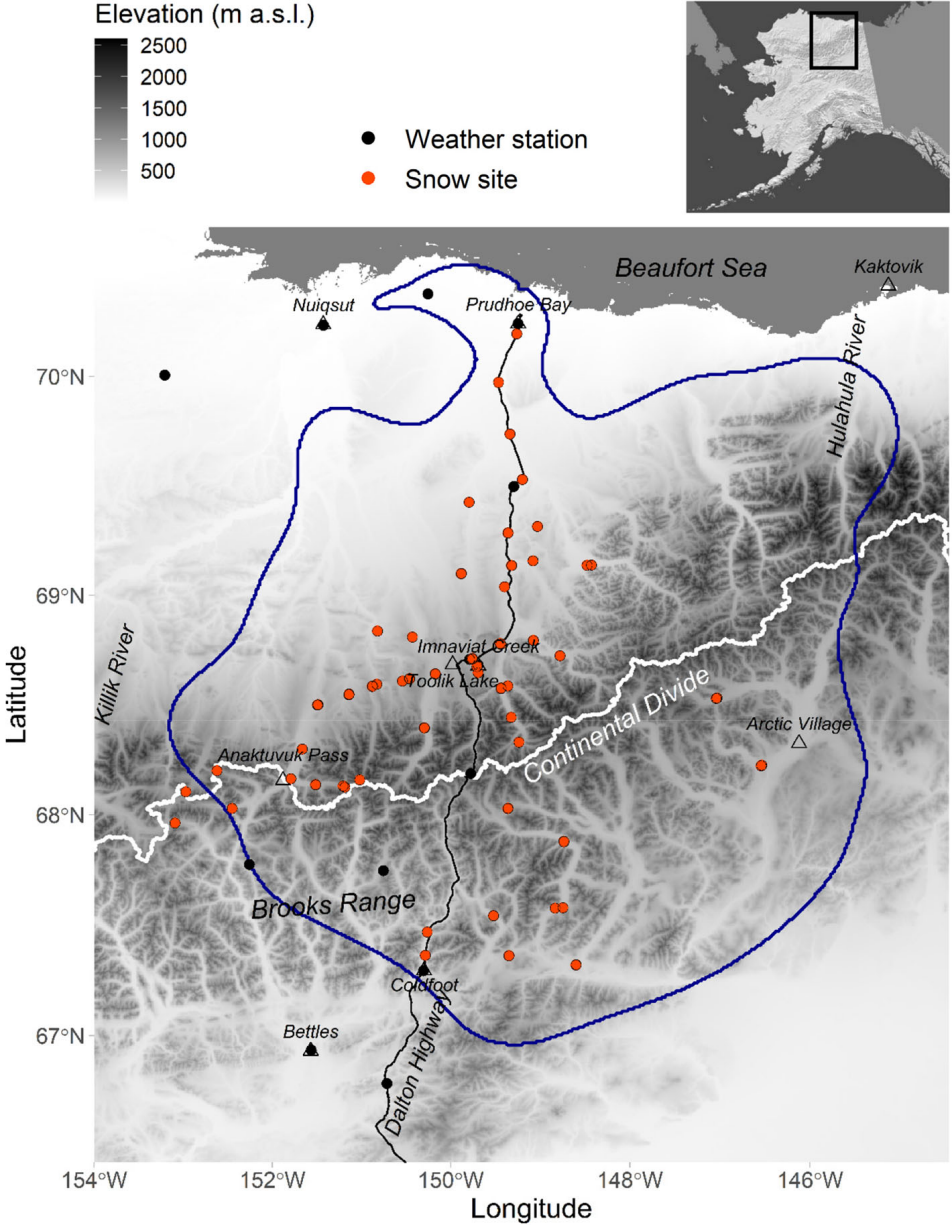
Map of the study area extending over the home range of the Central Arctic Herd (CAH) and including snow site locations (orange points) where snow measurements were made in March and April 2018, 2019, and 2020 by snowmobiles and fixed-wing airplane with skis; weather stations providing inputs to SnowModel (black points); a 95% kernel density estimate (KDE) based on CAH winter caribou distributions for the years 2001–2020 (blue polygon); the Continental Divide (CD; white line) of the Brooks Range (BR); the Dalton Highway (black line); and villages/monitoring sites (open triangles)

During the past four decades, the barren-ground caribou (*Rangifer tarandus granti*) of the Central Arctic Herd (CAH) in Arctic Alaska have been studied extensively, particularly in summer because their calving grounds are located in oil and gas extraction areas near the Beaufort Sea coast; (Fig. 1 ; [57–65]). The CAH population size was estimated at approximately 6000 animals in 1978 and grew steadily to its peak of 68,000 caribou in 2010 (with a slight decline during the early to mid-1990s), and was estimated to be roughly 30,000 animals in 2019 [60, 61, 66]. From their annual gathering on the coastal calving grounds in summer, CAH caribou generally disperse during early fall (~end of August – mid September) across the region extending from the Beaufort Sea coast, south to the Brooks Range (BR), and by October, they typically migrate further south to spend the winter either north or south of the Continental Divide (CD; running west to east along the crest of the BR; Fig. 1). For nearly two decades, from 2002 through 2020, Alaska Department of Fish and Game (ADFG) has annually monitored the general CAH winter distribution by estimating the proportion (%) of collared caribou located south of the CD in mid-March ([66–70]; Table 1). These historical surveys suggest that during the last 19 years, the majority of collared CAH caribou (≥50%) were distributed south of the CD in mid-March. In four of the 19 years, less than 10% of collared CAH caribou were located south of the CD, thus, in those years, the majority of the herd wintered north of the CD, typically in the northern BR foothills both east and west of the Dalton Highway (Table 1; Fig. 1; [57, 59, 67, 71–73]).

**Table 1:** Winter distribution (survey in mid-March by Alaska Department of Fish and Game; ADFG) of collared Central Arctic Herd (CAH) caribou south of the Continental Divide (CD) of the Brooks Range in winters 2001/02–2019/20 (Modified and updated from [66–70])

Winter	Year of mid-March ADFG survey	Number of collars located	% located ADFG (VHF and GPS) collars south of CD	% GPS-collared caribou in this study wintering south of CD	CAH primary winter range location, south (S) or north (N) of the CD
2001/02	2002	103	69	–	S
2002/03	2003	89	68	–	S
2003/04	2004	100	87	–	S
2004/05	2005	111	60	–	S
2005/06	2006	76	54	–	S
2006/07	2007	54	60	–	S
2007/08	2008	43	2	–	N
2008/09	2009	58	95	–	S
2009/10	2010	53	91	–	S
2010/11	2011	50	94	–	S
2011/12	2012^a^	10	80	–	S
2012/13	2013	39	100	–	S
2013/14	2014^a^	17	94	–	S
2014/15	2015	56	88	100	S
2015/16	2016	28	64	86	S
2016/17	2017	37	8	0	N
2017/18	2018	85	77	67	S
2018/19	2019	98	7	14	N
2019/20	2020	109	0	0	N

^a^ No ADFG tracking flights of VHF (very high frequency) radio collars were conducted in March when distribution of caribou can reflect winter distribution. Locations of global positioning system (GPS) and Platform terminal transmitter (PTT) satellite collars were recorded during the end of February in 2012 and end of March in 2014 to capture winter distribution

The winter ranges south of the CD include areas dominated by coniferous forest and areas of non-forested mountain slopes; this habitat type is hereafter called ‘mountainous boreal forest’. Winter ranges north of the CD generally include the broad, north-facing valleys and the northern foothills of the BR. The tundra habitat there includes wind-blown, rocky ridges, and rolling hills intersected by braided river deltas with shrub tundra and tussock tundra. Particularly during the six most recent winters (2014–2020), the CAH winter range location differed markedly from year-to-year between the tundra in the north and the mountainous boreal forest in the south (Table 1). We expected that an Arctic winter environmental attribute with the capability of changing dramatically from one year to the next influenced this winter-range variability, prompting our focus on evaluating the role of snow in CAH winter range selection. While we acknowledge that other climate variables, forage abundance, predation, disturbance, and neighboring herds’ winter range selection have been known to influence caribou winter range variability and winter movement, the effect of snow depth remains unquantified.

Specifically, we quantified the influence of snow depth on caribou winter range selection over a large region (~ 100s of kilometers) and evaluated the effect of snow depth and winter forage on daily movement at a landscape-scale (~ 100s of meters). Because this mountainous, Arctic Alaska study region is remote, nearly road-less, and highly inaccessible in winter, these investigations required a combination of snow field observations and snow modeling tools capable of representing the physics associated with snowpack evolution and distribution at regional and landscape scales to produce the necessary temporally and spatially explicit snow depth data. At the regional scale, these snow depth data were coupled with long-term CAH winter distribution records to study winter range location selection. At the landscape scale, these snow depth data and winter forage data were coupled with Global Positioning System (GPS) locations in animal movement models to quantify the effect of snow depth on daily caribou selection and movement rate (see animation of daily snow depth data and caribou location data in Additional file 1). We addressed the following questions:

1. Does snow depth encountered by CAH caribou in the fall correspond to observed difference in winter range location?
2. Once on winter range, how do snow depth and winter forage (lichen) abundance affect CAH caribou daily resource selection and movement rate?
3. Do the effects of snow depth on daily resource selection and movement rate differ between the two CAH wintering habitats: tundra north of the CD and mountainous boreal forest south of the CD?

Further, given the increased energy expenditure associated with movement through deeper snow [6], and the importance of lichen as a winter forage resource for barren-ground caribou [19, 44], we hypothesized that on a daily temporal scale, caribou (1) select for areas with lower snow depth than otherwise available in the surrounding landscape, (2) select for areas with higher lichen proportions than otherwise available in the surrounding landscape, and (3) caribou movement rates decrease with increasing snow depth. Finally, we expected this selection and movement behavior to be more pronounced when caribou winter in mountainous boreal forest than on tundra, because the boreal forest snowpack is typically deeper than the tundra snowpack.

## Methods

### Study area

Our study area encompassed all CAH GPS locations used in this study. It included parts of the traditional lands of Iñupiat, Nunamiut, Gwich’in, and Koyukon Athabascans, which is a section of the Brooks Range, the highest mountain range in the Arctic, as well as its foothills and the northern-most coastal plain of Alaska (Fig. 1). Specifically, the study area extended from the Beaufort Sea coast in the north, 460 km south to the southern foothills of the BR, and between the northward-flowing rivers, Killik River in the west, and the Hulahula River, 430 km to the east. Two main habitat types compose this area and were central to this research: tundra and mountainous boreal forest. The tundra vegetation is dominated by wet sedges, tussocks, and dwarf shrubs near the coast [74, 75], and sedges, shrubs, and tussocks in the northern foothills of the BR [76, 77]. The mountain slopes of the BR are dominated by low alpine tundra and barren or sparsely vegetated areas, while the valleys south of the CD are covered by more erect shrubs and coniferous boreal forest dominated by black spruce communities [76]. These two habitats are associated with two distinctly different types of seasonal snow cover: Arctic tundra snow is generally shallow (mean snow depth = 45 ± 21 cm; March – April 2018 and 2019 (this study) [78];) with windblown top layers that are likely capable of supporting a caribou without collapsing. In contrast, boreal forest snow is shallow to moderately deep (mean snow depth = 76 ± 17 cm; March – April 2018 and 2019 (this study); [78–80]) with a more fragile structure of weakly bonded, large snow grains (depth hoar), making it more likely that caribou sink deeper into the snowpack. The main reasons for these snowpack differences are: (1) a windier climate on the tundra resulting in increased redistribution of snow compared to the boreal forest, where the trees inhibit redistribution by wind; and (2) air temperature gradients across the region, defined by topographic elevation and distance to the coast and the ocean with variable sea-ice coverage [81–85].

### Snow data

#### Snow observations

To characterize the spatial snow depth variability within these two different snow types and between winters, we conducted six-week snow field campaigns March–April in 2018, 2019, and 2020 at 82 snow sites (Fig. 1; measurement protocols and snow site selection detailed by Pedersen et al. [78]). These 2018–2020 snow observations provided three winters of field-validation for the 2014–2020 snow depth datasets that were paired with caribou location data (see sections on SnowModel and Caribou data). The fieldwork on the tundra snow was completed by snowmobile traverses. The mountainous boreal forest snow sites were accessed by first landing on snow-covered lakes with a fixed-wing aircraft on skis and then traveling to sites on snowshoes. At each snow site, we measured snow depth at approximately every three meters along 200–800 m transects across the landscape using an automated snow-depth probe (MagnaProbe; [86]). We dug snow pits to identify snowpack stratigraphy and measured bulk and layer-specific snow density according to protocols detailed by Pedersen et al. [78]. To estimate a bulk snow water equivalent (SWE) at each snow site, we paired the bulk density (weighted by layer thicknesses) from each snow pit profile with the average snow depth measured in the nearby transects. Both site-specific SWE and density were assimilated in SnowModel (described in the section below). The individual snow depth measurements made along the transects were used for comparison with the modeled snow depths.

#### SnowModel

**Fig. 2:**
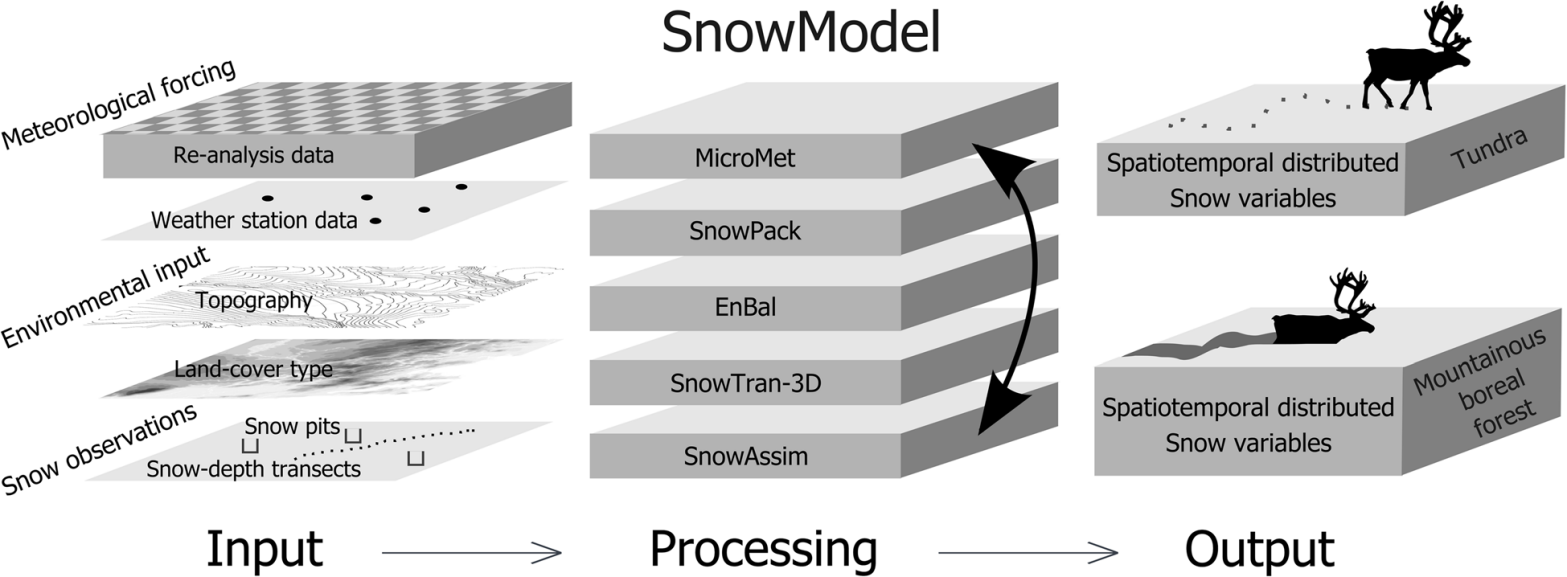
Schematic of SnowModel, including required input data (Input); coupling of MicroMet, SnowAssim, and the associated process and data assimilation models (Processing); and spatially and temporally explicit output data (Output)

To investigate the effects of snow depth on caribou winter range selection and winter movement patterns across space and time, we used snow depth data produced by SnowModel (Fig. 2; [22, 87]). This system allows users to generate fit-for-purpose snow information that is relevant for unique investigations of animal movement behaviors over appropriate, application-specific, spatial and temporal resolutions. For this application, we produced daily snow depth data at 90 m by 90 m spatial resolution, over the 430 km by 460 km study area (~ 200,000 km^2^; Fig. 1), for 1 September 2001–31 May 2020. The most relevant spatial resolution for each unique snow dataset should (1) resolve the physical processes that define the variability and distribution of the snow variable of interest across the landscape (e.g., snow redistribution by wind); (2) resolve the scale of the observed, dynamic movements of the study animal; and (3) balance (1) and (2) with the available computational resources while producing manageable output file sizes. We selected a 90-m spatial resolution because it resolved the variability in snow depth encountered over the approximate observed distance (~ 2 km) traveled by our wintering GPS-collared caribou in one day (i.e., our temporal resolution). The 90-m data resolved dominant, local, topography-specific snow-depth differences between valley bottoms and hilltops, and the low snow depth levels observed in the Brooks Range mountain valleys [88]. Lastly, these data dimensions were reasonable given our computational processing capabilities. SnowModel is the core of a suite of physically based snowpack-evolution, snow (re)distribution, and process modeling tools (Fig. 2; [22, 87]). Included in SnowModel is SnowAssim (Fig. 2; [89]), a submodel designed to assimilate field-based snow observations, such as snow density and SWE. SnowAssim works with SnowModel to simulate physics-based snow-landscape interactions and generate spatially and temporally distributed snow information between snow measurements in space and time. Through this assimilation of observed snow properties, the resulting snow information is physically realistic at measured and unmeasured locations and times across a landscape at user-defined spatial and temporal scales (e.g., [88, 90]). Hence, SnowModel provided spatially and temporally continuous distributions of snow properties that were consistent with our observational datasets.

A high-resolution meteorological model called MicroMet [91] provides meteorological data inputs to the SnowModel process models (Fig. 2). MicroMet spatially distributes meteorological information from local weather stations (in this application we used 15 weather stations; Fig. 1; [92, 93]) and gridded atmospheric reanalysis data (MERRA-2; [94]) over the study area. MicroMet requires data for five variables: air temperature, relative humidity, precipitation, wind speed, and wind direction.

SnowModel inputs also included two temporally invariant environmental data layers (Fig. 2): the National Elevation Dataset [95] was used to describe topography, and North American Land Change Monitoring System (NALCMS; [96]) provided land-cover data to define the spatial distribution of land-cover types across the region. The topography dataset is used by MicroMet to define temperature and precipitation lapse rates and wind speed acceleration and deceleration across the landscape [91]. This information, in turn, is used to define snow evolution features such as snowfall and snowmelt distributions, and the redistribution (i.e., transport) of snow by wind. The land-cover information defines the variability in vegetation heights used in SnowModel; the heights are used to control whether snow is available for redistribution by wind or is, instead, immobilized by vegetation [97, 98]. We generated our required snow depth datasets by running SnowModel and SnowAssim for a 19-year simulation, while assimilating snow density and SWE observations (together these define snow depth; [99]) when available across the study area and period. For 2001–2017, we used SWE data from the Imnaviat Creek snow monitoring site in the northern BR foothills (Fig. 1; [100, 101]), and for 2017–2020, we used SWE and density observations from our field expedition/traverse datasets (Fig. 1; [78]).

Our field snow-property observations [78] and other historical snow-free date observations [102] provided evidence for a consistent, relatively thin or non-existent snow cover in the major northward-facing valleys of the BR, north of the CD. During our field campaigns, we observed the greatest number of caribou foraging in these valley areas, and Nicholson et al. [57] also observed a frequent use of these valleys in earlier years (2003–2007). High wind speeds in these valleys are responsible for increased sublimation and wind-erosion of the snowpack, leaving them relatively snow-free during winter compared to the surrounding landscape [103]. To ensure our models accurately reproduced the observed shallow snow depth and high wind speeds in these valleys, we developed a wind-speed correction mask using annual snow-free date datasets for the region [102]. This adjustment was required because the weather stations used to provide meteorological inputs to SnowModel (Fig. 2) were located too far from these valleys to detect and record these locally increased wind speeds. In the tundra areas covered by the correction mask, the wind speed was increased in SnowModel by a factor of 1 to 7. The largest correction (factor 7) was applied to wind speed in these major north-facing valleys, while wind speed in areas between the valleys received little to no adjustment. This correction served to increase snowpack wind erosion and sublimation in these northward-facing valleys and was required for SnowModel and SnowAssim to reproduce our observed snow depths.

### Winter forage data

Lichen constitutes a dominant proportion (60–70%) of barren-ground caribou winter diet (e.g., [19, 44]). Lichen species occur throughout the CAH winter range [46, 104], and we detected lichen types (*Cladonia* spp. and *Cetraria* spp.) below the snow in 65% of our snow pits during fieldwork in 2017–2020 (S. H. Pedersen 2020, unpublished data). The patchy distribution of lichen makes mapping of its spatial distribution and biomass challenging. While recent methodologies have been used to successfully map lichen distributions at the landscape scale (~ 30 m; [105–108]), large portions of the CAH winter range are not yet covered by these lichen abundance datasets. Therefore, we mapped caribou winter forage across our study area using the proportion of lichen-rich vegetation based on the 30-m NALCMS land-cover map [96]. The lichen proportion was calculated as a continuous variable, and defined as the fraction of the nine 30-m by 30-m grid cells (i.e., three grid cells by three grid cells or a 90 m by 90 m area total) classified as erect shrub tundra or alpine tundra (non-tussock) vegetation, which are both defined as containing a minimum of 20% lichen cover [96]. This lichen-proportion dataset produced values that ranged from 0.0 to 1.0; higher lichen proportions (i.e., 0.78–1.0) were more common north of the CD than south. In this study, the proportion of lichen was assumed to be constant through time.

### Caribou data

#### Caribou capture and monitoring

During the months of March, April, or June in years 2001–2019, adult (≥2 years old) female and male caribou were captured by ADFG for collaring using a hand-held net-gun fired from a low-flying helicopter (model R-44, Robinson Helicopter Co., Torrance, California). Caribou were fitted with either Very High Frequency (VHF) or GPS equipped satellite collars (Telonics, Mesa, AZ, USA).

We used caribou GPS location data representing male (11) and female (65) caribou collected over nearly six years (September 2014–May 2020, excluding the months of June, July, and August; Table 2). To obtain equal time intervals (fix rates) throughout the winter season for each individual, we resampled all caribou locations at various fix rates (2, 8, and 12 h) to produce a complete time series from fall to spring using the Animal Movement Tools (amt) R package [109]. The resampling yielded three location datasets of 8-hourly, 12-hourly, and daily location frequency, where 8- and 12-hourly fix rates existed for only a subset of the monitored animals and varied in availability throughout our six study years (Table 2). While daily location data represents the coarsest temporal resolution applicable in many types of movement analyses [110], we chose to use daily locations (24-h fix rates) for the analyses, because it was the fix rate resulting in the highest number of individuals with adequate data throughout the winter season (Table 2), i.e., providing the maximum number of individuals for our analyses.

**Table 2:** Number of GPS-collared CAH caribou included in the movement analyses, and average winter-specific movement rates ±1 standard deviation (SD) in kilometers per day (km/d; also for 8-h and 12-h fix rates so that the values are comparable)

Fix rate (hours)	Winter	Number of GPS-collared caribou	Number of locations	Average movement rate ± 1 SD (km/d)
24	2014/15	16	3303	2.9 ± 1.0
24	2015/16	14	2626	2.6 ± 0.6
24	2016/17	15	2164	3.1 ± 1.2
24	2017/18	43	6353	1.8 ± 1.1
24	2018/19	28	4752	2.7 ± 0.9
24	2019/20	21	2673	2.2 ± 0.6
12	2014/15	0	0	–
12	2015/16	0	0	–
12	2016/17	0	0	–
12	2017/18	30	10,741	2.1 ± 1.6
12	2018/19	20	7183	2.9 ± 1.6
12	2019/20	16	4387	2.7 ± 1.3
8	2014/15	10	5343	3.1 ± 1.3
8	2015/16	11	5591	2.9 ± 1.3
8	2016/17	10	5460	3.7 ± 1.5
8	2017/18	12	5962	2.8 ± 1.3
8	2018/19	8	4377	3.3 ± 1.5
8	2019/20	5	4891	1.5 ± 0.9

#### Fall snow depth

The CAH caribou typically experience the first snowfall in early fall (August–September) and the bulk of the tundra snowpack is established during the ‘early cold’ season of sustained, below-freezing air temperature, usually late September through early December [111]. The typical early-fall distribution of the CAH is from the Beaufort Sea coast to the northern foothills of the BR (e.g., [67, 71–73]). To evaluate whether the CAH use snow depth encountered across this area during early fall as a winter-range selection cue, we used all available GPS locations for the month of September (8-h, 12-h, and 24-h fix rates in years 2014–2019; *n* = 43,680) to represent this scattered, early fall distribution. Located north of the BR, this distribution extended from 153.9°–146.0° W and from 68.4°–70.2° N (Fig. 1). To generate an annual average fall snow depth, we extracted the daily, modeled snow depth in these GPS locations from 1 September through 31 December and averaged all values per year 2001/02–2019/20. To evaluate the relationship between long-term, observed CAH winter range location (i.e., ADFG mid-March distributions that we assumed approximate their winter range location north or south of the CD; Table 1) and average fall snow depth from 2001/02–2019/20, we used two methods. We first used a simple Pearson’s correlation test to estimate the correlation between the two variables. Secondly, we used a mixed-effects logistic regression with binomial errors [112] to conduct a deeper examination of the relationship between these two variables and how they may have changed over time. The mixed-effects logistic regression used the total number of individuals detected during annual ADFG surveys that wintered south of CD and the total number of individuals detected during annual surveys that wintered north of CD (Table 1) as the response variable and the annual value of fall snow depth and a linear year trend as continuous explanatory variables. The linear trend in wintering location with year was included to test whether the wintering location changed consistently over the study period. We included random intercepts for each year to account for the interannual variability of the 19 years and to account for overdispersion caused by unexplained variability, likely due to other factors that arguably contribute to the herd’s winter-range selection (see the Discussion section) but are not included in our model. Hence, our mixed-effects logistic regression included fall snow depth and a linear year trend as fixed effects and random intercepts for individual years. We calculated odds ratio (i.e., *e*, the natural logarithm base, raised to the exponent of the model estimate per explanatory variable, *e*^estimate^) to evaluate the effect of the variables included in the model [112]. We used the ‘lme4’ R package for these statistical analyses [113].

### Movement modeling

#### Data preparation

In preparation for our movement analyses, we identified missing data in our CAH caribou location time-series dataset, and placed missing values (NA) at times when a location should have been recorded but was missing. GPS collars are programmed to record a location at a fixed hour, but the location dataset can contain imprecisions, because the actual time of location recording may vary seconds to minutes from that specified hour. Because a constant time lag between successive location measurements was required for our movement analyses, we rounded the times based on a reference date (e.g., with the hour 00:00), so that our locations were exactly 24 h apart. As a consequence of rounding the timestamps, an individual’s position may also need to be corrected; for example, if the time of a location is corrected to occur 30 min later than the actual recording occurred, then the position of the individual also needs a correction, which is made under the assumption that an animal moves forward with a constant speed [114]. We used the functions ‘setNA’ and ‘sett0’ in the R package adehabitatLT to regularize our trajectories [115].

To objectively identify the wintering period (i.e., determine when animals were on winter range) for each GPS-collared caribou in each year (defined as the date at which each caribou arrived on winter range through the date that it departed winter range), we first calculated the movement rate for each relocation of each animal. We then used the method of Lavielle [116, 117] to partition the animal trajectory into segments characterized by homogenous behavior, in this case, sustained, relatively low movement rates. We identified a maximum of ten segments for each winter, with each segment containing a minimum of ten relocations. Typically, for each individual, this method identified four general segments per year, during the time 1 September–31 May: (1) A period during early fall (prior to fall migration) of intermediate movement rates (~ 5 km per day), where the CAH caribou typically inhabited the coastal plain and the region north of the BR; (2) a short-term period of fall migration with abruptly elevated and maximized movement rates (up to ~ 50 km per day); (3) following the fall migration, a period of minimum, sustained, relatively low winter movement rates (~ 1.8–3.1 km per day; Table 2; this low-movement rate period was typically the longest; ~ 4–7 months, and we selected this longest segment as the winter range period for each individual for the analyses of this study); (4) finally, for individuals with GPS locations available throughout April and/or May, the final segment of elevated movement rates (up to ~ 50 km per day) was identified and defined as spring migration. Six individuals had inadequate data and were excluded from the analyses. The total number of individuals used in the analyses is in Table 2. All preparation of the GPS location data and movement analyses were done in R version 3.6.0 [118].

#### Integrated step selection analysis

We used integrated step selection analysis (iSSA) [119] to test the hypothesis of whether movement patterns of wintering caribou were influenced by snow depth and the proportion of lichen-rich vegetation in tundra or mountainous boreal forest winter habitats, i.e., north and south of the CD. Additionally, elevation was tested as a potential covariate because of the topographic differences between these two habitat types and its likely importance for caribou movement. Step selection functions [110, 120] are widely applied in animal movement ecology to investigate resource selection at the spatial and temporal scale of a movement step, which is defined to be a relocation of an animal between two points: a starting point and endpoint at fixed time intervals. These analyses operate under the assumption that locations closer to an animal are more likely to be used than locations farther away [121, 122]. By fitting a conditional logistic regression model, step selection functions account for the fact that for each observed step/relocation (i.e., used point), there exists alternate locations (i.e., available points), unique to that observed time, that the animal could have selected instead [120]. Therefore, these analyses allow relatively fine-scale assessment of selection as an animal moves through the landscape. We randomly generated ten available points for each used point; the locations of available points were based on each animal’s individual distributions of observed turning angle and step length (the Euclidian distance traveled per day) from their own used steps recorded during each individual winter. Snow-depth and lichen-proportion covariate values were extracted at the endpoint of each used and available step to describe the snow depths and lichen proportions that caribou moved towards, in order to test these covariates' effect on selection. In addition to the characteristics of a step selection function, iSSA allows the user to include movement parameters, such as turning angle and step length, in the regression model, thus producing an estimate of movement and selection parameters in the same model [123]. We included the interaction between snow depth and step length as a covariate in the iSSA to quantify whether snow depth had a significant effect on caribou movement rate (i.e., step length). For this covariate, we used snow depth values that were extracted from our spatially and temporally continuous snow depth dataset at the starting point of each step, rather than the endpoint to evaluate how the initially encountered snow depth influenced subsequent step length, i.e., daily movement rate.

Each of the six winters in this study were categorized according to the primary winter distribution defined by where the majority of the collared caribou (≥50%; Table 1) were wintering. We grouped the three winters 2016/17, 2018/19, and 2019/20, when the CAH primarily wintered on tundra north of the CD and grouped the three winters 2014/15, 2015/16, and 2017/18, when the CAH primarily wintered in mountainous boreal forest south of the CD. We conducted the movement analyses with the caribou location data grouped by this primary winter distribution, and identified each step by individual and winter. Consequently of this habitat-specific (tundra vs. mountainous boreal forest) grouping of the winters, all GPS-collared caribou (Table 2) were included in the movement modeling, i.e., contributed to the population estimates of selection coefficients, despite some of those caribou wintered elsewhere than the habitat designation of a given winter (*n* = 2 in 2015/16, *n* = 14 in 2017/18, *n* = 4 in 2018/19, Table 1). An iSSA was fitted to each individual caribou per winter with the resulting estimates averaged (non-weighted) to produce a population-level estimate per winter range location north and south of the CD (using the R package amt; [109]). To facilitate easier comparison of effect sizes (i.e., the relative strength of selection) between covariates, the snow depth and lichen proportion values used in the iSSAs were centered by subtracting their mean and were then scaled by dividing the centered values by their standard deviation.

The iSSA results were reported as *relative selection strength* and *probability of use*, following methods and recommendations by Avgar et al. [124]. We conducted separate iSSAs using 8-hourly, 12-hourly, and daily location data for the two winter ranges. To avoid collinearity [123, 125] between covariates included in iSSAs, we generated pair-wise Pearson’s correlation coefficients (*r*) for all variables (snow depth, lichen proportion, and elevation), and considered variables with *r* ≥ 0.6 to be highly correlated [126]. If two variables were highly correlated, we only retained the most informative variable in our model. We included only snow depth and lichen proportion in the iSSAs; we excluded elevation because it was significantly correlated with snow depth (*r* ≥ 0.6) in two of the six winters (*r =* 0.33, 0.65, 0.72, 0.30, 0.31, 0.52 for 2014/15–2019/20, respectively).

## Results

### Snow observations

**Fig. 3:**
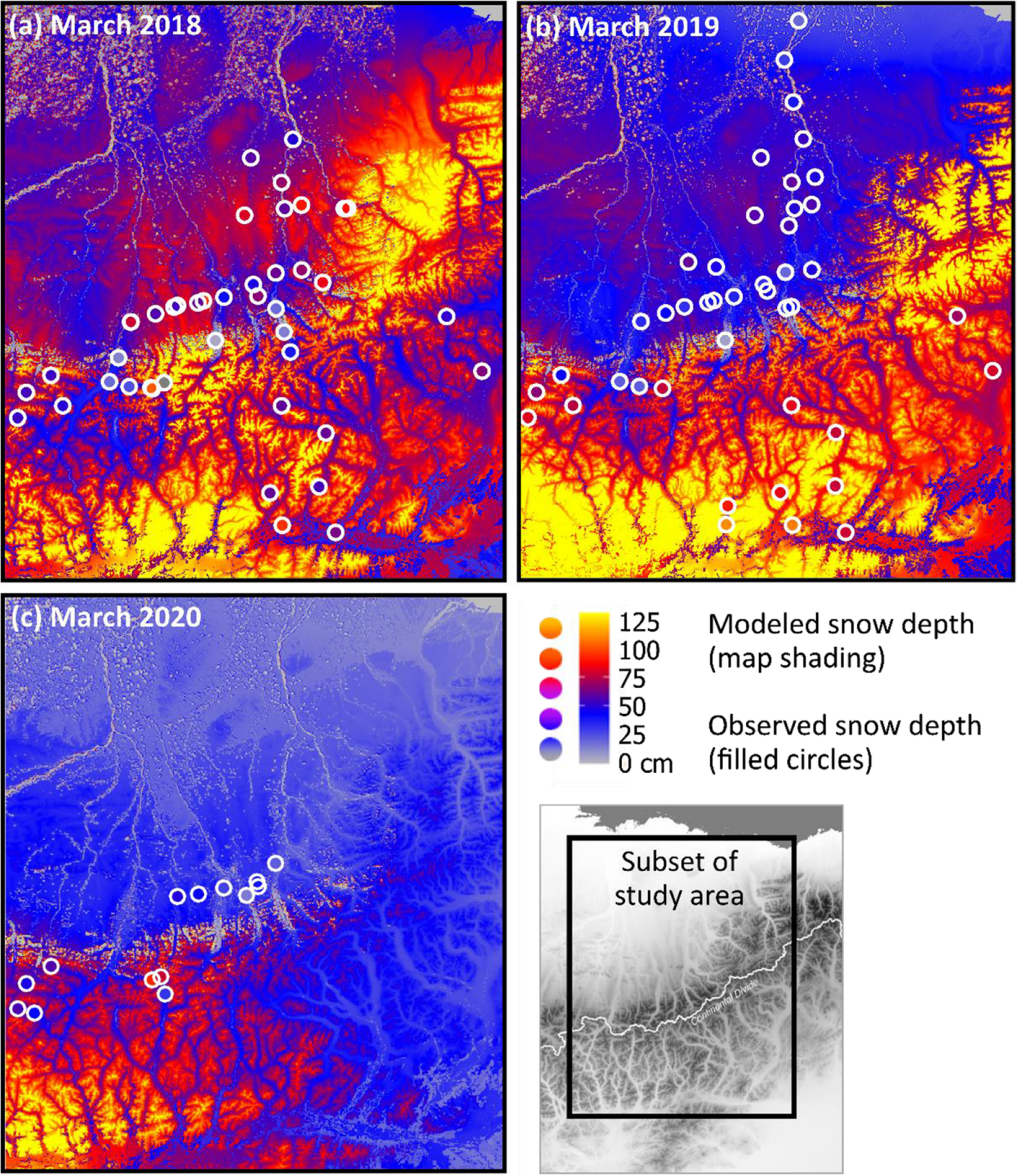
SnowModel modeled snow depth (cm; map shading) on 15 March (**a**) 2018, (**b**) 2019, and (**c**) 2020, and observed mean snow depths at snow sites visited from 6 March through 5 April in 2018, 2019, and 2020 (filled circles). The color scales of modeled and observed snow depths are identical

**Fig. 4:**
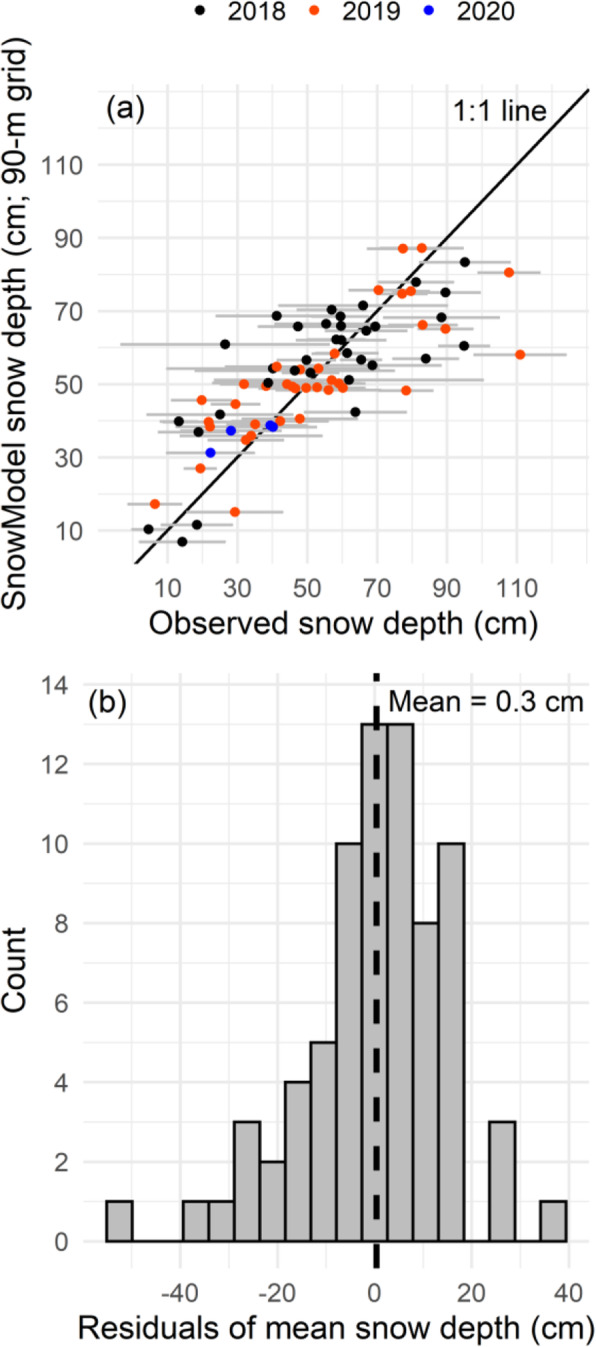
**a** Modeled mean snow depth plotted against mean snow depth observations at snow sites in March and April 2018 (black), 2019 (orange), and 2020 (blue), the horizontal error bars are ±1 standard deviation of observed snow depth, and the 1:1 line is black. **b** Residuals of modeled and observed mean snow depth (modeled-observed; cm) with the mean residual (0.3 cm) marked with a dashed line

Our snow observations collected in 2018, 2019, and 2020 included bulk SWE, snow density, and more than 1500 snow depth measurements at the 82 snow sites (2018 *n* = 17,743; 2019 *n* = 10,091; and 2020 *n* = 1543; Fig. 1). The SnowModel and SnowAssim regional snow depth distributions across our study area described the observed, spatial snow-depth variability among snow site locations (Fig. 3a, b, c; the map shading is modeled snow depth and the filled circles are average snow depth per snow site). The average snow depths ranged from 5 cm to 111 cm (Fig. 4a; the axes scales show the snow-depth range among snow sites and the grey ±1 standard deviation error bars show the variability of observed snow depth within each snow site). The modeled snow depths extracted at transect locations at each snow site were significantly correlated with snow depths observed along those transects for each snow site during the three years, based on Pearson’s correlation coefficients (*r* = 0.76 (2018); *r* = 0.80 (2019); *r* = 0.87 (2020); Fig. 4a). Generally, the range in modeled snow depth captured the observed range with a mean residual of 0.3 cm between mean modeled and observed snow depth (Fig. 4b).

### Fall snow depth and winter range selection

**Fig. 5:**
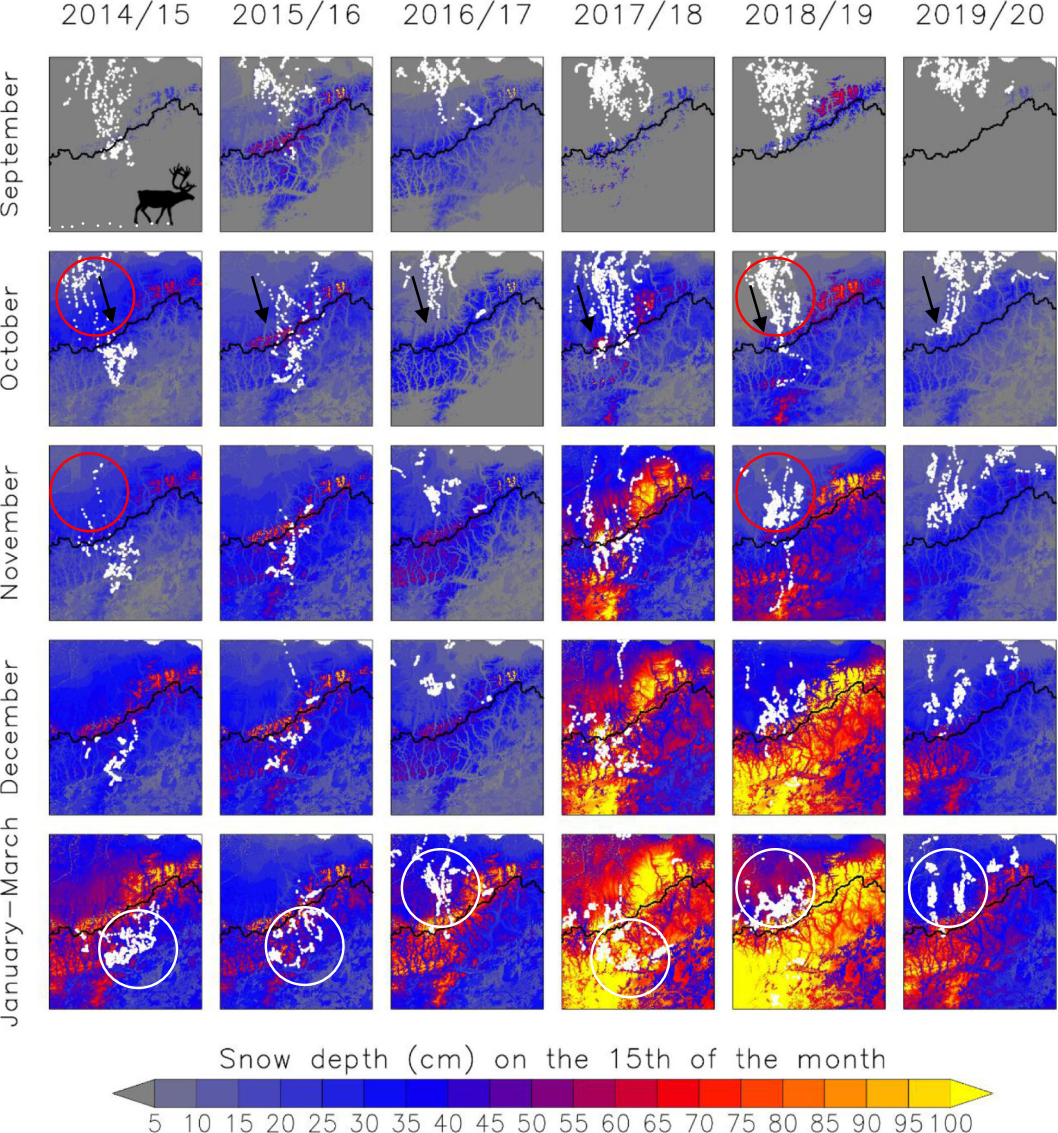
Monthly snow depth (cm; grey to yellow shades) and CAH daily GPS locations (white points; Table 2) per month for September, October, November, and December, and January–March for the winters 2014/15–2019/20. SnowModel snow depth is from the 15th of each month and the caribou locations for the entire month are shown for September–December. The January–March panels display all GPS locations from 1 January to 31 March and the snow depth distribution on 15 March. The black arrows in October panels mark the southward migration direction and the primary annual winter range location is marked with a white circle in the January–March panels. Red circles mark the approximate area of fall snow depth difference between October–November 2014 and 2018. The black line marks the Continental Divide of the Brooks Range

In early fall, the CAH is typically distributed north of the CD. The timing of the first snow accumulation in this area coincided with the timing of CAH caribou selection of winter range location (Fig. 5; animation in Additional file 1). The first day on the winter range (i.e., when exhibiting wintering behavior of relatively low, daily movement rate) for GPS-collared individuals of this study was between 7 October and 7 December. Because a substantial portion of the snow cover is typically formed during the months September–December (Fig. 5), the snow depths that caribou encounter at that time are generally a consistent predictor of the snow depths they will experience during the subsequent winter months (*r* = 0.88, *p* < 0.001). Though the fall snowpack evolution does vary across our large study region and between years [85, 127], a potential explanation for this predictive quality of the fall snow depth is that often a wind event during the fall months creates a wind slab on top of the snowpack. This compact top layer/crust can act as a lid and prevent the underlying snowpack layers from being eroded by wind transport during the remainder of the winter, i.e., the snowpack will remain at an approximately similar depth after this fall wind event [85].

The CAH distribution and movement varied throughout the winters 2014/15–2019/20 (Fig. 5). In September, GPS-collared CAH caribou were distributed from the Beaufort Sea coast south to the BR (Fig. 5); these early-fall distributions were similar across the six years. The fall migration, identified as more directed movements, was initiated in October during most years. The GPS data indicated that by November and December, caribou reduced their movement, i.e., exhibited wintering behavior, and remained in a final winter range location (Fig. 5). We evaluated whether fall snow depth (September–December average) in the area north of the CD (marked by red circles in Fig. 5), acted as a cue for caribou to either move south through the BR to winter in mountainous boreal forest, or stay on the north side of the CD to winter primarily on tundra. Two winters that clearly exemplified both cases were 2014/15 and 2018/19. The deeper average snow depths (~ 20–35 cm) north of the CD in October–November 2014 were followed by movement to winter ranges primarily south of the CD, while shallower average snow depths (~ 0–15 cm) in October–November 2018 were followed by use of winter ranges north of the CD. After arrival on the winter range, most GPS-collared caribou remained in that general location until March, or when they initiated spring migration (Fig. 5).

**Fig. 6:**
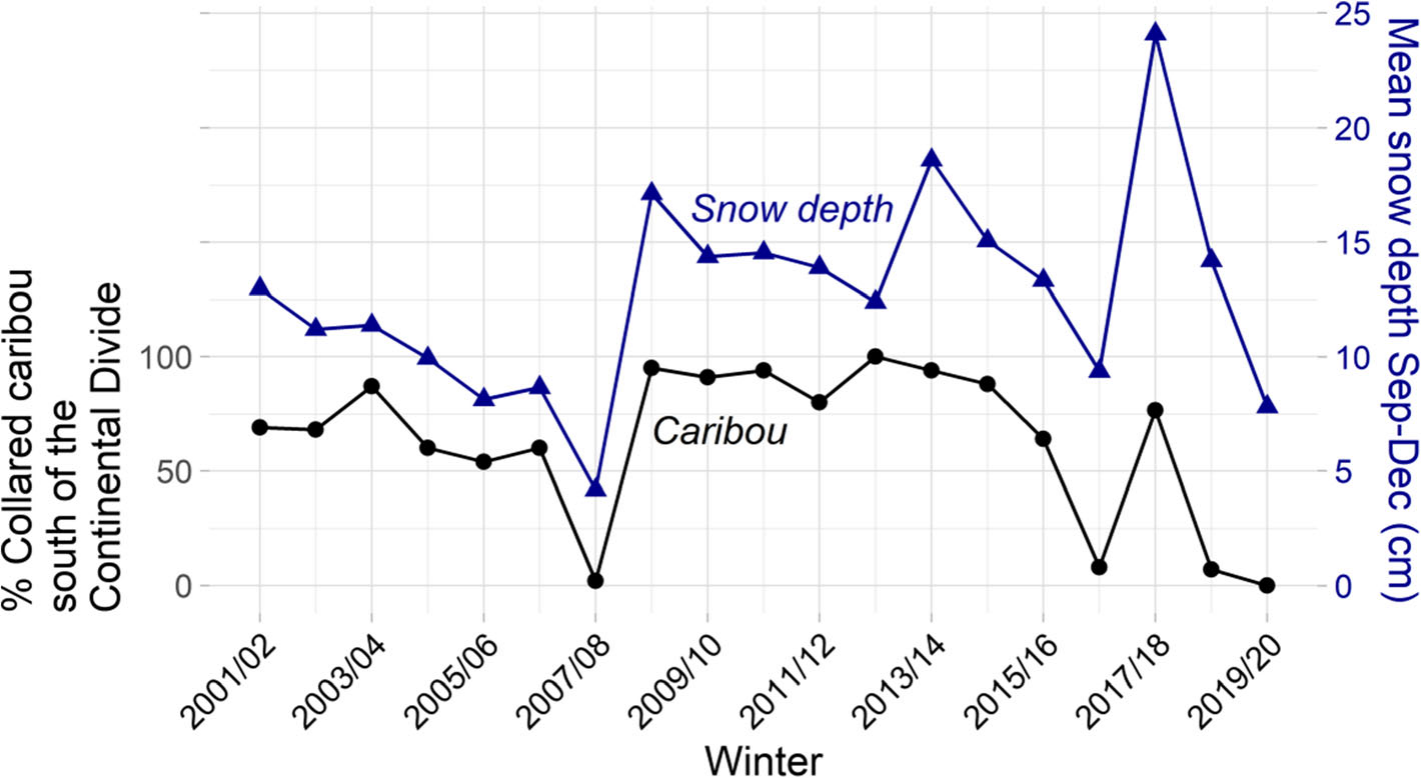
Proportion (%) of Alaska Department of Fish and Game (ADFG) collared caribou wintering south of the Continental Divide in February–March 2001–2019 (Table 1; [66–70]; black filled circles, left y-axis). Snow depth averages (cm) for the September–December period over the range of GPS-collared animals 2014/15–2019/20 (i.e., the area occupied by the animals prior to fall migration) north of the Continental Divide (blue triangles, right y-axis). There is a statistically significant correlation between the average fall snow depth and the winter location (Pearson’s correlation coefficient, *r* = 0.59, *p* = 0.008)

The monthly and annual distribution of caribou provides a visualization of the ways that snow depth may play a role in defining CAH caribou selection of winter range (Fig. 5). During years with relatively shallow fall snow depths north of the CD, which typically equated to lower-snow winters overall, the majority of the CAH stayed north of the CD (e.g., winters 2016/17, 2018/19, and 2019/20; Fig. 6). Conversely, deeper fall snow depths north of the CD, likely signaling a snow-rich winter, corresponded with the majority of CAH caribou wintering south of the CD (as was observed in 2014/15, 2015/16, and 2017/18; Fig. 6). During fall seasons with intermediate snow depths, a large proportion, but not all, of the collared CAH animals wintered south of the CD, as they did during years with deeper fall snow depths (Fig. 6). The modeled snow depths were used to quantify this relationship. Our results indicated that fall snow depths correlate strongly with CAH winter range locations during 2001/02–2019/20 (Fig. 6; Table 1), yielding a significant positive correlation between winter range location and fall snow depth (*r* = 0.59, *p* = 0.008, *n* = 19). The modeled estimates of fall snow depth and linear trend in wintering location with year in the mixed-effects logistic regression model (including random intercepts per year) were both statistically significant. These results confirmed the Pearson’s correlation test result; the modeled estimate of change in wintering location with fall snow depth (estimate 0.39; 0.22 and 0.58, lower and upper 97.5% confidence interval [CI], respectively) was significantly different from zero. This result indicates that proportionately more CAH caribou winter south of the CD in years with higher mean fall snow depth. In other words, a single unit (= 1 cm) increase in fall snow depth increases the odds of wintering south of the CD by a factor of 1.5 (1.2 and 1.8, lower and upper odds ratio 97.5% CI, respectively), when holding all other variables constant. Furthermore, the proportion of caribou wintering south of the CD declined over time. However, within this annual trend in winter distribution, fall snow depth exerted a strong influence on the winter range selection. Based on examination of the residuals, the annual pattern in winter distribution was largely driven by an abrupt change in later years; caribou were more likely to winter south of the CD in years before 2015/16, than in the years after 2015/16.

### Snow depth effects on movement rate and selection

In addition to evaluating the relationship between fall snow depths and CAH caribou winter range location, we tested the hypotheses of whether snow depth affects movement rates and selection once the caribou were on winter range. We extracted daily, in situ snow depths and lichen proportion associated with caribou GPS locations (Fig. 5) for the 2014/15–2019/20 winters. The annual average snow depths at the endpoints of both available and used steps ranged from 22 ± 16 cm to 43 ± 29 cm (mean ± SD) in winters primarily spent north of CD (2016/17, 2018/19, and 2019/20) and from 35 ± 15 cm to 64 ± 28 cm in winters primarily spent south of CD (2014/15, 2015/16, and 2017/18).

**Fig. 7:**
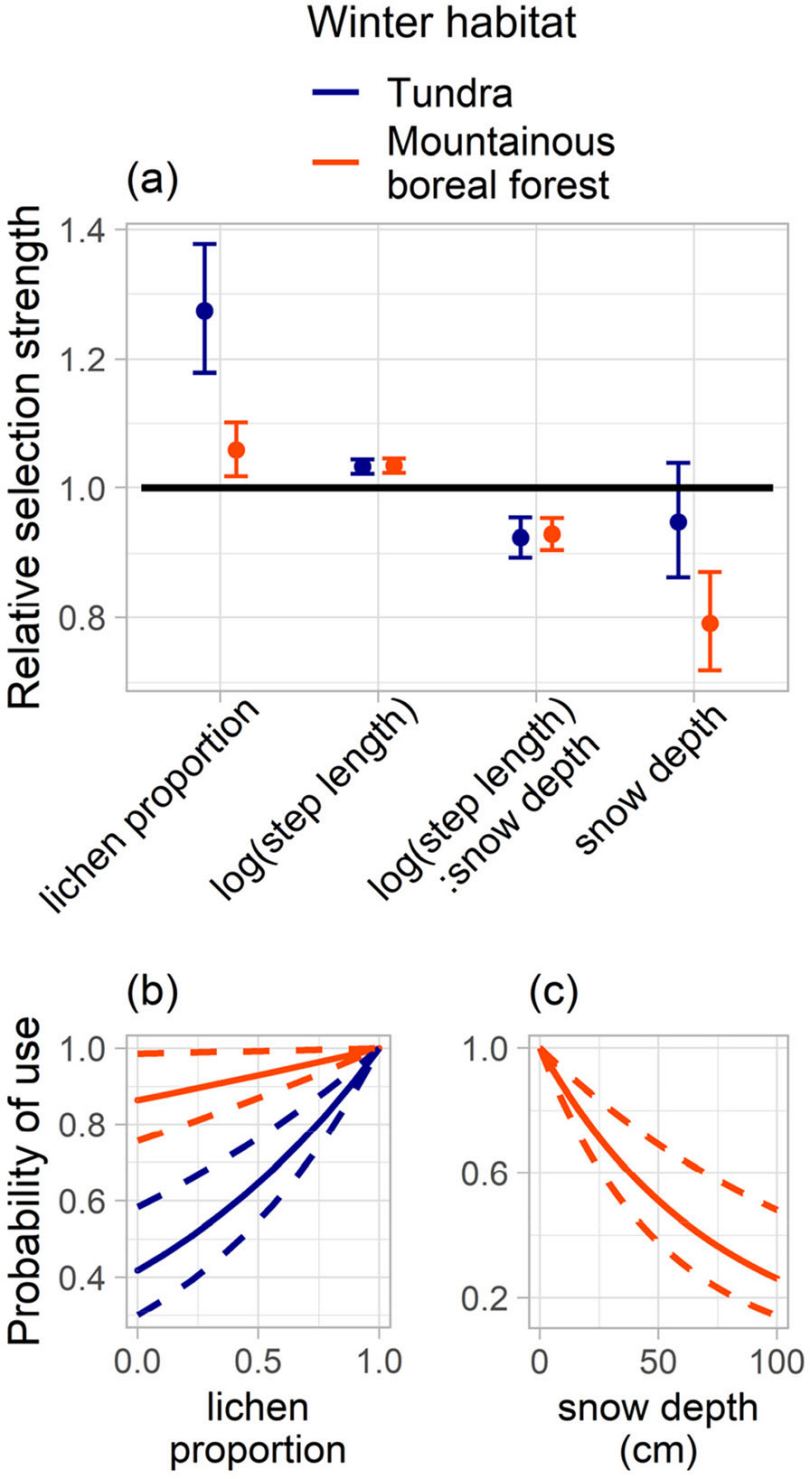
**a** Relative selection strength [124] of population-level averages of integrated step selection analysis (iSSA; [119]) coefficient estimates for winters CAH caribou spent primarily on tundra (*n* = 114,631; blue points and 95% confidence interval (CI) error bars) and in mountainous boreal forest (*n* = 136,554; orange points and 95% CI error bars). Covariates in the iSSA model are log(step length), i.e., movement rate; the interaction between step length and snow depth in the starting point of a step (log(step length):snow depth); lichen proportion and snow depth at the endpoint of a step. Coefficient estimates greater than 1.0 (above the solid black line) indicate selection while estimates lower than 1.0 (below the solid black line) indicate avoidance. Coefficient estimates with 95% CI error bars that do not overlap the 1.0 line indicate that this relationship is statistically significant. The coefficient estimates below 1.0 for log(step length):snow depth indicate an inverse relationship between step length and snow depth. The winter habitats tundra (winters 2016/17, 2018/19, and 2019/20; blue) and mountainous boreal forest (winters 2014/15, 2015/16, and 2017/18; orange) roughly correspond to north and south of the CD, respectively. **b** Probability of use with varying lichen proportion when wintering on tundra (blue lines; dashed lines are 95% CI) and in mountainous boreal forest (orange lines; dashed lines are 95% CI). **c** Probability of use with varying snow depth (orange dashed lines are 95% CI) for winters spent in mountainous boreal forest. There was no statistically significant effect of snow depth on movement in winters on tundra. The probabilities are based on mean coefficients of iSSA only including snow depth and lichen proportion at the endpoint of a step (not scaled or centered datasets)

Our iSSA results revealed that snow depth affected CAH caribou movement and selection on the winter range, but the quantified relationship differed between tundra and mountainous boreal forest habitats. During winters when the CAH primarily remained north of the CD on tundra, at the scale of our analyses (90-m and daily data), snow depth was not a statistically significant driver of caribou selection (Fig. 7a). Therefore, caribou did not display positive or negative selection for snow depth, indicating neither selection for nor avoidance of deeper snow. In contrast, in years when the CAH primarily wintered in the mountainous boreal forest south of the CD, caribou showed an increased probability of use with decreased snow depth (Fig. 7c) indicative of negative selection for snow depth, i.e., avoidance of areas with deeper snow (Fig. 7a).

To account for the role of forage in caribou winter selection patterns, we also included lichen proportion in the iSSA models. The presence of lichen-rich habitat (i.e., lichen proportion = 1.0) was higher north than south of the CD; in endpoints of available and used steps, high lichen proportions occurred more frequently for winters primarily spent north of CD (2016/17, 2018/19, and 2019/20) than south (2014/15, 2015/16, and 2017/18). During all six winters, regardless of winter range habitat type, GPS-collared caribou selected for areas with higher lichen proportions, as indicated by a statistically significant positive selection coefficient (Fig. 7a). Probability of use increased in areas with a higher lichen proportion in both the tundra and mountainous boreal forest environments (Fig. 7b). We found no statistically significant correlation between snow depth and lichen proportion for the six winters (*r =* − 0.047, − 0.24, − 0.37, − 0.18, − 0.37, − 0.095 for 2014/15–2019/20, respectively). Hence, at this scale of analysis and using our lichen proportion dataset, the lichen abundance was not significantly higher in areas with shallow snow. These results suggest that caribou movement rate and selection within the winter range is driven independently by snow depth and lichen proportion.

Finally, iSSA results suggested that on winter ranges both on tundra and in mountainous boreal forest, caribou movement rates were significantly and inversely proportional to the snow depths encountered at the starting point of each daily step (Fig. 7a). Hence, caribou movement rates were significantly lower when animals experienced greater initial snow depths. These results (based on daily location data from 2014 to 2020) were consistent with the iSSA results from separate analyses using 8-hourly and 12-hourly location data. Furthermore, the average movement rates were significantly different and higher for caribou wintering primarily north (2.6 km per day) than south (2.1 km per day; *t* (74) = 2.5, *p* = 0.01) of the CD.

## Discussion

The importance of snow is widely recognized in Arctic wildlife research, and snow information that is relevant and appropriate to wildlife applications is of great interest [21]. While the role of snow in caribou winter ecology has been observed and discussed as being important in movement and definition of winter range characteristics (e.g., [57, 128]), research that integrates snow and animal location data to quantify the direct impacts of snow depth on this snow-adapted species has been rare. This is mainly due to the lack of snow data at wildlife-relevant spatiotemporal scales (e.g., hourly to daily and meters instead of kilometers resolutions) and the lack of wildlife-relevant snow property information (e.g., snow depth vs SWE, which is often inaccurately used as proxy for snow depth). To overcome these issues and incorporate germane snow-property data at appropriate scales, we used snow depth datasets that spanned the entire CAH fall and winter range; these datasets were produced at sufficient spatial and temporal resolution to account for local-scale snow-depth variability and evolution to match the spatiotemporally dynamic character of caribou winter movement. Combined with multiple years of CAH locations, these snow depth datasets enabled us to evaluate how caribou adjusted their regional winter range location, and day-to-day movement patterns once on winter range, in response to regional and local snow depth distributions, respectively.

### Fall snow depth as a cue to winter range location

Mountains present little obstacle to migratory caribou [19, 129] and CAH caribou are no exception. In 15 of the recent 19 winters (2001/02–2019/20), the majority of collared CAH caribou migrated through and over the BR mountains to access winter ranges south of the CD (Fig. 5; Fig. 6; Table 1), though the specific winter ranges varied longitudinally among winters (this study; [57, 58, 67]). In that context, the four winters 2007/08, 2016/17, 2018/19, and 2019/20 that the CAH spent primarily north of the CD represent an abrupt change to a long-term pattern of using southern winter ranges. The ADFG survey (Table 1) showed that prior to and including winter 2015/16, the CAH winter range selection was more consistent among consecutive years, but after 2015/16, the selection became more variable among years, and the herd tended to winter north of the CD more frequently. This selection pattern may explain the significant linear trend in winter range selection reported in the mixed-effects logistic regression results. However, in the years after 2015/16 the fall snow depth still seemed to exert a strong influence on winter range selection. Such abrupt deviations from one year to the next are also documented in the neighboring Teshekpuk Caribou Herd (TCH) and other Arctic caribou herds [130, 131]. We found a strong significant correlation (*r* = 0.59, *p* = 0.001) between the fall snow depth and the proportion of collared caribou wintering south of the CD, and while this relationship was not completely driven by extreme snow years, snow depth had a significant effect on CAH winter range selection. While both our correlation test and logistic regression results suggest that caribou respond to fall snow depth, it is important to emphasize that fall snow depth levels reported herein (< 25 cm) are considered a *cue* and may have limited biological significance, e.g., in inhibiting caribou travel given their relatively long legs and efficient locomotion in relatively deep snow [6, 19]. However, from a snow-science perspective, fall snow depth is likely to be a reasonable *predictor* of snow levels that may inhibit movement or forage access in subsequent winter months (January–March) (*r* = 0.88). North of the CD, the majority of the snowpack is typically established in September–December [85, 111]. Therefore, fall snow depth is often a reasonable predictor of snow-depth levels throughout the remaining winter.

Snow acting as a trigger or cue for migration and winter range selection has been reported previously for the CAH. Roby [36] noted that, following the first snow in the fall, groups of CAH caribou made a southward movement into the BR, while others stopped at the northern edge of the foothills. Similar examples exist for other Alaska caribou herds, e.g., an extraordinarily heavy August–September snowfall near the Arctic coast and in Anaktuvuk Pass in 1960 and 1961 triggered migration of Western Arctic Herd (WAH) caribou south of the BR [34]. In interior Alaska, the Denali Herd caribou made an unusual movement 200 km north of their regular winter range following a record September snowfall in 1992 [132]. In Canada, Le Corre et al. [133] found that caribou arrived earlier on winter range in years with early fall snow. They suggested that caribou use the amount of October precipitation to predict snow abundance during migration, and that animals adjust their migration timing to limit the energetic costs of moving through deep snow [133]. Our temporally and spatially explicit snow data can be used in individual-based quantification of such potential cueing effects of snow depth (or other derived snow variables, e.g., rate of accumulation, timing of first snowfall, depth variability, and snow-coverage of forage species) and other relevant environmental variables produced by SnowModel (e.g., air temperature, wind speed, wind direction, and day length) on caribou winter range selection and migration timing.

In reality, migratory caribou winter-range selection is influenced by a suite of environmental and biological factors and herd-specific demographic parameters (e.g., population size, herd density, and sex and age structure; [47, 134, 135]). The importance of these other factors may vary between years and between herds, and they may act together in complex ways. While our data are coarse (i.e., annual surveys with varying sample sizes and areal average snow depth) and our analyses focus solely on estimating the role of fall snow depth in winter range selection over 19 years, these analyses highlight the importance of snow in influencing winter range selection. Furthermore, our results demonstrate that snow should be included in future analyses, along with a range of other factors documented as being influential for caribou wintering location, e.g., overgrazing of slow-growing lichen species [42, 136]. Macander et al. [108] reported that caribou in interior Alaska prefer lichen habitats in winter until they become overgrazed, and consequently, the herd shifts wintering area within the boreal forest region. The CAH winter range variation may be explained by consistent use of winter ranges south of CD from 2001/02 to 2015/16, interrupted only when the very low fall snow depths allowed caribou to winter north of CD in 2007/08 (Fig. 6). It is possible that by 2016/17, the southern winter ranges could have been overgrazed and the caribou began wintering north, except that they were ‘pushed’ south by record snow in 2017/18 (Fig. 6). The lichen proportion dataset used in our study is invariable in time and provides no information on lichen biomass, and it is therefore inadequate to test this overgrazing hypothesis. Because the TCH, the Porcupine Caribou Herd (PCH), and occasionally the WAH may utilize parts of the same winter ranges as CAH [39, 61] these neighboring and/or larger caribou herds could also exacerbate overgrazing south of the CD.

Although not included in our analyses, predator avoidance may be an additional factor influencing CAH winter range selection, since predation pressure is likely different between the two general wintering areas. During snow-free months, wolves (*Canis lupus*), grizzly bears (*Ursus arctos*), and golden eagles (*Aquila chrysaetos*) prey on CAH caribou, but in winter, wolves are expected to be their primary predator [137, 138]. Wolf surveys report higher wolf densities in the BR mountains (6 wolves per 1000 km^2^; [137]) than further north on the tundra of the coastal plain (2–4 wolves per 1000 km^2^; [139]). Further research is needed to understand how forage availability and predation pressure, and their interaction with snow, may affect the CAH selection of winter range location and local movement once on winter range.

Finally, herd size is suggested to impact winter range variability; the larger the herd, the larger the required winter range [57, 129, 140]. During the period 2008–2013, when the CAH was near a recent maximum size (estimated population size > 50,000 animals; [67]), the majority of the herd (≥80% of collared animals; Fig. 6, Table 1) wintered south of the CD, which supports this supposition. However, according to both the strong correlation between winter distribution and fall snow depth and our mixed-effects modeling results suggesting that fall snow depth acts as a cue for winter range selection, and the fact that the winters 2008/09–2012/13 had fall snow-depth levels north of the CD above average for our 19-year time series (> 12.7 cm; Fig. 6), snow depth is also a likely explanation. Moreover, in winter 2017/18, the CAH size was estimated to be ~ 23,000 animals, a recent low, yet most of the collared caribou wintered south of the CD that winter, while fall snow depths were at a record high level on the north side of CD. We propose that the herd’s selection of southern winter ranges may be triggered by relatively deep fall snow depths *in addition* to herd size. While Nicholson et al. [57] mapped CAH winter range location and discussed potential drivers of its year-to-year variability, they recommended further research of environmental factors such as inter-annual differences in winter weather and snow cover. Here we investigated one possible mechanism, suggesting that fall snow depth is an important cue, and may likewise be important for caribou and reindeer winter range selection elsewhere in the Arctic outside of our Alaska study domain.

### Snow depth and lichen affect caribou movement within winter range

Early scientific accounts describe caribou in Alaska as year-round ‘wanderers’, with directed movements occurring only during fall and spring migration [7]. The mid-winter months are characterized by the least amount of nomadism, and caribou reduce their movement rate to the lowest annual levels during this time (this study; [34, 38, 57, 141, 142]). Our results show that once CAH caribou have selected their winter range, they remain in the same general area until March or the initiation of spring migration (Fig. 5). This pattern is consistent with observations made of other neighboring caribou herds [19, 39, 142].

#### Decreased movement rate with increasing snow depth

This minimal winter movement may be caused by snow levels. Murie [7] observed that heavy snowfall can act as a movement barrier for wintering caribou in Alaska. Our results suggest that both on tundra and in mountainous boreal forest, CAH caribou move more slowly when in areas of deeper snow (Fig. 7a). The 24-h fix rate of our GPS locations does not allow for identification of different daily activities (e.g., moving, feeding, resting). However, the daily winter activities of PCH caribou have been found to vary by season, day length, and snow conditions, and these daily activities were dominated by feeding and resting [19]. Russell et al. [19] found that the least time was spent moving (running, trotting, and walking) and most time was spent lying (ruminating) and feeding during periods of adverse snow conditions, including increased snow depth. These observations of decreased movement and increased lying and feeding intensity can translate to an overall lower movement rate (i.e., shorter distance traveled per day) in deep snow, which our results support. Further, based on our knowledge of this snow-covered landscape, its differential snowpack properties (tundra vs. mountainous boreal forest snow), and informal observations of the caribou behavior and movement in these landscapes during our field campaigns, we speculate that there are several plausible explanations for the decreased movement rate with increasing snow depth (Fig. 7a), and that these are different for the mountainous boreal forest and tundra winter habitats.

In the mountainous boreal forest, a snowpack of more than 70 cm deep can reduce caribou mobility [143] and increase energetic costs, likely because this depth threshold exceeds their approximate chest height (~ 50 cm; adult domesticated reindeer; [49]) or leg length (~ 50–60 cm; adult CAH caribou; hind foot length; [144]). In our mountainous boreal forest snow sites, average snow depth ranged from 50 ± 8 cm to 111 ± 13 cm (March/April 2018 and 2019 average snow depth = 76 ± 17 cm for all mountainous boreal forest snow sites, *n* = 18), and the average modeled snow depth of 49 cm suggests that many of the starting locations of used steps exceed or are near the snow-depth threshold for impeding caribou movement. These observations support the iSSA result of decreased movement rates when caribou encountered increased snow depths. Caribou may choose to remain in a smaller area due to the higher energetic costs of movement in areas of deep snow. Such reduction in movement rate with increased snow depth is also observed for PCH caribou during October in the central BR mountains [35], where the CAH and PCH winter ranges overlap [61]. Conversely, the iSSA result can also be interpreted as *increased* movement rate with *decreasing* snow depth. On the winter ranges south of the CD, the snow depth is relatively shallow in tree-less alpine tundra areas or on lakes large enough for the wind to erode the snowpack. This relatively shallow snow may promote fast movement, for example, to avoid predators. Finally, it takes more time to crater through deep snow than shallow snow. Hence, feeding caribou in the relatively deep snow of the mountainous boreal forest often stay longer, dig one large crater, and move less than in areas with shallow snow [19].

On the tundra, the snow depths are generally more shallow (average 45 ± 21 cm for all tundra snow sites; *n* = 53; [78]) and present a limited impeding effect on movement, particularly in the frequently-used, wind-blown north-facing valleys with shallow snow depth (5 ± 5 cm – 22 ± 10 cm; *n* = 5; [78]). Furthermore, the GPS-collared caribou moved significantly faster in winters spent primarily north of the CD on tundra, than when south of the CD. Hence, the *increased* movement rate with *decreasing* snow depth may be the most plausible explanation for the iSSA result for tundra (Fig. 7a). Though, in more sheltered areas of the otherwise windy tundra environment, e.g., areas between the broad, wind-blown valleys, the snow was relatively deep (ranging 42 ± 13 cm – 90 ± 10 cm; *n* = 5; [78]). Our snow observations show that these areas experienced limited wind erosion, thus the snow there is both softer and deeper, and possibly capable of hindering caribou movement because of these properties. Finally, the generally shallow tundra snowpack also allows faster feeding, faster movement, and the ability to travel longer distances, because less time is spent digging and cratering [19].

#### Selection of snow depth

Our results further suggest that CAH caribou wintering south of the CD in the mountainous boreal forest selected for areas with less snow than was available in the surrounding area (Fig. 7a, c). Arctic Alaska caribou are often found on wind-blown ridges and mountain slopes, where forage is more readily available because of snow-removal by wind [7]. While they do not hesitate to descend into the spruce forest and to crater through considerable snow depositions for lichen [19, 143], caribou wintering in boreal forest systems tend to select feeding sites in more shallow snow [51]. Significant selection of more shallow snow depths may also be explained by the inherent characteristic of the boreal forest snowpack; it is typically fully comprised of relatively large, weakly bonded snow grains (depth hoar). When cratered by caribou, the structure of the snowpack fails and the disturbed snow subsequently metamorphoses into a more dense and well-bonded snow cover [145, 146]. Hence, reworking relatively deep snow that has previously been cratered and solidified is more energy consuming, and for this reason the likelihood of reusing previously cratered snow is low [49]. Selecting areas with shallow snow for feeding may also be advantageous for energy conservation [12]. The hardening process of cratered and disturbed snow also occurs in tundra snow, but the areal extent of undisturbed and shallow snow makes this a negligible factor on selection within the tundra landscape. This may explain our result showing that GPS-collared caribou did not show any significant selection for snow depth during winters spent north of the CD on the tundra.

#### Selection of lichen proportion

The wandering nature of caribou is undoubtedly influenced by the snow depths they encounter during winter, but the scattered spatial distribution of lichen [108] may also contribute to their movement behavior. We found that, irrespective of winter range location, caribou selected for areas with higher lichen proportion than otherwise available in the surrounding landscape, indicating the importance of this winter forage resource (Fig. 7a, b). This result agrees with observations by Roby [36], who found that lichen was consistently selected by the CAH caribou in feeding areas, despite its sparse distribution. The highest relative selection strength for lichen proportion was seen in CAH caribou wintering north of the CD (Fig. 7a), where fruticose lichen is abundant on surfaces where the wind has eroded the snowpack [36]. The effects of both forage distribution, and the snow depth on the accessibility of that forage, are considered to be related and inseparable factors influencing caribou winter movement and foraging behavior and should be assessed together [32, 35, 36]. While the dataset of distributed lichen proportion applied in our movement models does not provide information on lichen biomass, and admittedly represents a coarse measure of winter caribou forage abundance, this study is one of few studies quantifying the combined effects of snow depth and winter forage on caribou movement. New methodologies for mapping and modeling distributions of lichen and other caribou forage species are emerging [105–108, 147], however, these improved lichen and forage distribution estimates are not available across our entire study area. Future research using such data, e.g., in combination with SnowModel snow depth and caribou location data, could help quantify the effects of snow depth on caribou winter forage accessibility by cratering (e.g., [50, 148]). Key to such investigations are location data of sufficient temporal detail to determine activity states (e.g., foraging/cratering, running, walking, resting/ruminating) of each individual (e.g., [149, 150]). Hence, our results are likely the first of many investigations to combine snow and forage data to advance our understanding of caribou winter foraging behavior across space and time.

### Perspectives

We acknowledge that, in addition to snow depth, other aspects of the snowpack can be critical to caribou winter ecology. The strength of the snow surface and individual snow or ice layers buried within or below the snowpack is likely important for caribou locomotion and foraging accessibility in both tundra and mountainous boreal forest wintering habitats. Quantitative models of strength properties of snow and ice across space and time do not yet exist, and essential to their development is the ability to represent the direct effects of ice layers formed by, e.g., rain-on-snow (ROS) and mid-winter melt-freeze events [151]. ROS events are still rare in Arctic Alaska, but predicted to increase in frequency [152], and have previously caused a major change in the TCH winter distribution [131, 152]. Furthermore, ROS events are hypothesized to account for the majority of PCH population fluctuations [64], and the icing resulting from ROS events influence ungulate forage access [153, 154]. Across the Arctic, investigations of the relationships between climate variables, including some winter and snow-related parameters, and long-term time series of caribou and reindeer life-history event observations (e.g., calving date and timing of migration), indicate that winter processes are highly influential factors (e.g., [155, 156]). We encourage further investigation of the underlying mechanisms linking snow to caribou life-history events to understand the effects that snowpack depth and strength can exert on caribou nutritional condition through, e.g., regulating forage accessibility and the energetic expense of locomotion. Both are likely to have consequences for caribou fitness at the individual and population level through parameters including survival, parturition or birth rate, recruitment of calves to the herd, or reproduction timing [157–159]. Spatially and temporally explicit snow information such as the data acquired and used in the research presented herein are highly applicable to such investigations, because they enable a quantification of and accounting for: (1) inter-seasonal carry-over effects (e.g., [158, 160]); (2) short-lived weather events that have significant long-term population effects (e.g., mid-winter icing or ROS events [64, 161, 162]); (3) snow phenology throughout winter (i.e., temporal evolution of the snowpack and timing of snowfall events [163]); and (4) differential effects of snow on caribou and their predators (e.g., [164]). Additionally, such studies would benefit from interdisciplinary research teams of snow and wildlife professionals collaboratively working to gain a more complete understanding of the aspects of caribou ecology dependent on, and influenced by, snow.

## Conclusions

Across Arctic Alaska, a long history of use, dependence, and research, through observations (e.g., [7, 129]) and Traditional Ecological Knowledge (e.g., [165, 166]), has laid the foundation for our current understanding of caribou ecology. In our research, we integrated methodologies from snow and wildlife sciences. We used SnowModel and field observations to provide snow depth data that resolved the snow-depth variability across both the entire CAH home range and at a more detailed scale representing the snow-depth distributions caribou encountered on a day-to-day basis. We found that fall snow depth on the north side of the CD likely is an important cue that either, if shallow, motivates CAH caribou to stay on the tundra north of the CD, or if deep, promotes migration to winter ranges in the mountainous boreal forest south of the CD. When wintering south of the CD, caribou avoided areas with deeper snow and selected areas with higher lichen abundance. Both on the tundra and in the mountainous boreal forest, caribou movement rates decreased with increased snow depth. For snow properties, this research focused solely on snow depth, but we anticipate that numerous other snow properties are key in governing wildlife movement and health. Using SnowModel in future investigations will enable answering increasingly complex questions involving other snow properties (such as strength), icing events, forage accessibility, and winter mobility that will greatly advance our knowledge of caribou winter ecology.

## Supplementary Information

**Additional file 1:** Animation showing daily, modeled snow depth (background map shading) produced using SnowModel from 1 September 2018–31 May 2019 (dates will iterate at the top of the animation) overlaid by daily Central Arctic Herd caribou GPS-locations (white circles).

**Additional file 2: **R scripts to reproduce Fig. 4, Fig. 6, and Fig. 7 and the movement modeling using integrated Step Selection Analysis.

## Data Availability

The datasets generated and analyzed during the current study are available and permanently archived at NSF Arctic Data Center: Stine Højlund Pedersen. 2021. Observed and modeled snow depth in Arctic Alaska 2001–2020. Arctic Data Center. 10.18739/A2WM13V3Q. R scripts generated during this study are included in Additional file 2. The caribou GPS location datasets analyzed during the current study are not publicly available due to Alaska law [AS 16.05.815(d)]. These laws allow the release of such information to parties that have been authorized to perform specific activities as long as the parties agree to use the information only for purposes as provided under an agreement with the Alaska Department Fish and Game, contact Elizabeth A. Lenart.

## Abbreviations

ADFG: Alaska Department of Fish and Game
BR: Brooks Range
CAH: Central Arctic Herd
CD: Continental Divide
GPS: Global Positioning System
h: Hour
iSSA: integrated step selection analysis
KDE: kernel density estimate
km/d: Kilometer per day
NALCMS: North American Land Change Monitoring System
PCH: Porcupine Caribou Herd
ROS: Rain on snow
SD: Standard deviation
SWE: Snow water equivalent
TCH: Teshekpuk Caribou Herd
VHF: Very high frequency
WAH: Western Arctic Herd
