# Supplementary material for: Quantifying effects of snow depth on caribou winter range selection and movement in Arctic Alaska
Source: Mov Ecol. 2021 Sep 22;9:48. doi: 10.1186/s40462-021-00276-4 (PMC8456671; doi:10.1186/s40462-021-00276-4)
Supplement: Supplementary file 2 — Additional file 2: R scripts to reproduce Fig. 4, Fig. 6, and Fig. 7 and the movement modeling using integrated Step Selection Analysis. [file 40462_2021_276_MOESM2_ESM.zip › Pedersen_et_al_Description_Additional_file_2.docx]

**Additional file 2**

**R scripts used in:**

Pedersen, S. H., Bentzen, T. W., Reinking, A. K., Liston, G. E., Elder, K., Lenart, E. A., Prichard, A. K., Welker, J. M. (2021): Quantifying effects of snow depth on caribou winter range selection and movement in Arctic Alaska. Movement Ecology. DOI: 10.1186/s40462-021-00276-4.

**Content:**

R-scripts to reproduce figures 4 and 6 (Fig. 1–3, and 5 are visualizations and contain no analyses):

Fig. 4: *Pedersen_et_al_Figure_4.R*

Fig. 6: *Pedersen_et_al_Figure_6.R*

R-scripts to reproduce the movement modeling using integrated Step Selection Analysis (iSSA):

*Pedersen_et_ al_iSSA_procedure_Figure_7a_May2021.R*

*Pedersen_et_ al_iSSA_procedure_Figure_7bc_May2021.R*
